# Supplementary material for: Modified Sol–Gel Synthesis of Mesoporous Borate Bioactive Glasses for Potential Use in Wound Healing
Source: Bioengineering (Basel). 2022 Sep 5;9(9):442. doi: 10.3390/bioengineering9090442 (PMC9495454; doi:10.3390/bioengineering9090442)
Supplement: Supplementary file 1 [file bioengineering-09-00442-s001.zip › bioengineering-1879949-supplementary.pdf]

Table S1: The calculated kinetic of B-ions releasing during 168 h of incubations.

B					
**Time**	Ag 0	Ag 1	Ag 2.5	Ag 5	Ag 7.5
**0-24**	2.33333333	2.833333	3	3.333333	3.75
**24-72**	3.20833333	3.708333	2.458333	2.8125	3.041667
**72-168**	-1.25	-0.58333	-1.04167	-1.09375	-1.15625
**Average**	1.43055556	1.986111	1.472222	1.684028	1.878472
**Increase compared to MBG-Ag 0**	-	38.83495	2.912621	17.71845	31.31068

Table S2: The calculated kinetic of P-ions releasing during 168 h of incubations.

P					
**Time**	Ag 0	Ag 1	Ag 2.5	Ag 5	Ag 7.5
**0-24**	-0.20833333	-0.41667	-0.29167	-0.20833	-0.25
**24-72**	-0.10416667	-0.125	-0.10417	-0.08333	-0.08333
**72-168**	-0.125	-0.10417	-0.125	-0.14583	-0.09375
**Average**	-0.14583333	-0.21528	-0.17361	-0.14583	-0.14236
**Increase compared to MBG-Ag 0**	-	47.61908	19.04765	0	-2.38093

Table S3: The calculated kinetic of Ca-ions releasing during 168 h of incubations.

Ca					
**Time**	Ag 0	Ag 1	Ag 2.5	Ag 5	Ag 7.5
**0-24**	1.04166667	1.416667	0.875	0.791667	0.666667
**24-72**	0.20833333	0.145833	0.145833	0.083333	0.166667
**72-168**	0.1875	0.125	0.083333	0.114583	0.052083
**Average**	0.47916667	0.5625	0.368056	0.329861	0.295139
**Increase compared to MBG-Ag 0**	-	17.3913	-23.1884	-31.1594	-38.4058

Table S4: The calculated kinetic of Na-ions releasing during 168 h of incubations.

Na					
**Time**	Ag 0	Ag 1	Ag 2.5	Ag 5	Ag 7.5
**0-24**	0.41666667	0.666667	0.916667	1	0.875
**24-72**	0.04166667	0.125	0.041667	0.125	0.125
**72-168**	0.04166667	0.020833	0.03125	0.0625	0.145833
**Average**	0.16666667	0.270833	0.329861	0.395833	0.381944
**Increase compared to MBG-Ag 0**	-	62.5	97.91666	137.5	129.1667

Table S5: The calculated kinetic of Mg-ions releasing during 168 h of incubations.

Mg					
**Time**	Ag 0	Ag 1	Ag 2.5	Ag 5	Ag 7.5
**0-24**	0.33333333	0.5	0.958333	1.041667	1
**24-72**	0.125	0.166667	0.083333	0.166667	0.270833
**72-168**	0.13541667	0.135417	0.135417	0.125	0.114583
**Average**	0.19791667	0.267361	0.392361	0.444444	0.461806
**Increase compared to MBG-Ag 0**	-	35.08772	98.24561	124.5614	133.3333

Table S6: The calculated kinetic of K-ions releasing during 168 h of incubations.

K					
**Time**	Ag 0	Ag 1	Ag 2.5	Ag 5	Ag 7.5
**0-24**	0.33333333	0.625	0.75	0.958333	1.125
**24-72**	0.16666667	0.125	0.166667	0.229167	0.291667
**72-168**	0.13541667	0.15625	0.21875	0.364583	0.395833
**Average**	0.21180556	0.302083	0.378472	0.517361	0.604167
**Increase compared to MBG-Ag 0**	-	42.62295	78.68852	144.2623	185.2459

Table S7: The calculated kinetic of Ag-ions releasing during 168 h of incubations.

Ag					
**Time**	Ag 0	Ag 1	Ag 2.5	Ag 5	Ag 7.5
**0-24**	-	0.083333	0.25	0.5	0.791667
**24-72**	-	0.041667	0.0625	0.125	0.0625
**72-168**	-	0.052083	0.09375	0.114583	0.125
**Average**	-	0.059028	0.135417	0.246528	0.326389
**Increase compared to MBG-Ag 1**	-	-	129.4109	317.6455	452.9391

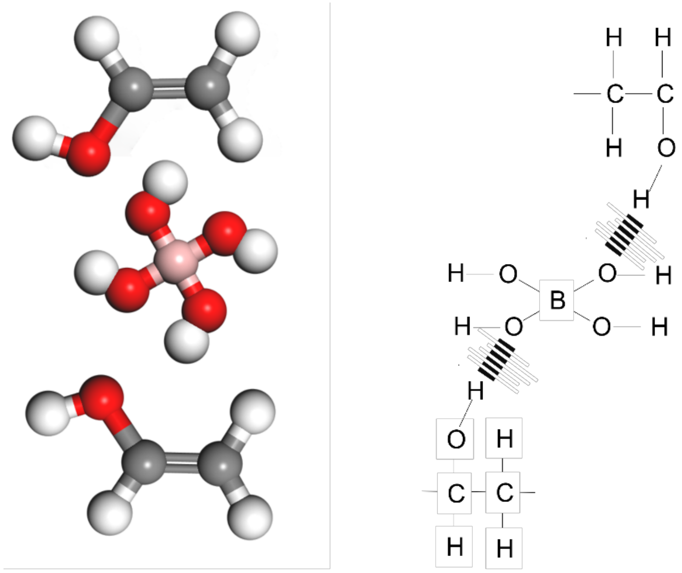

Figure S1: The structure of poly vinyl borate which forms during the synthesis process.

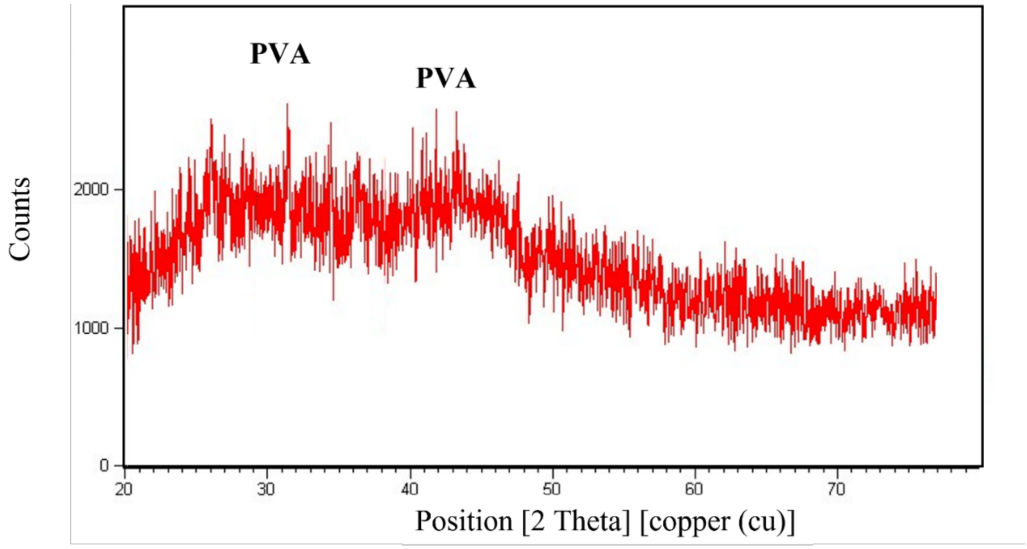

Figure S2: The XRD pattern of the formed gels after this in-situ deposition of compounds.
